# NAADP-evoked Ca^2+^ signals through two-pore channel-1 require arginine residues in the first S4-S5 linker

**DOI:** 10.1016/j.ceca.2017.09.003

**Published:** 2017-12

**Authors:** Sandip Patel, Dev Churamani, Eugen Brailoiu

**Affiliations:** a Department of Cell and Developmental Biology, University College London, London WC1E 6BT, UK; b Center for Substance Abuse Research, Lewis Katz School of Medicine at Temple University, Philadelphia, PA 19140, USA

## Abstract

Two-pore channels (TPCs) are two-domain members of the voltage-gated ion channel superfamily that localize to acidic organelles. Their mechanism of activation (ligands such as NAADP/PI(3,5)P_2_ versus voltage) and ion selectivity (Ca^2+^ versus Na^+^) is debated. Here we report that a cluster of arginine residues in the first domain required for selective voltage-gating of TPC1 map not to the voltage-sensing fourth transmembrane region (S4) but to a cytosolic downstream region (S4-S5 linker). These residues are conserved between TPC isoforms suggesting a generic role in TPC activation. Accordingly, mutation of residues in TPC1 but not the analogous region in the second domain prevents Ca^2+^ release by NAADP in intact cells. Our data affirm the role of TPCs in NAADP-mediated Ca^2+^ signalling and unite differing models of channel activation through identification of common domain-specific residues.

## Highlights

• The S4–S5 linker of TPCs harbours conserved basic residues.
• Arginine residues in the S4–S5 linker are required for activation of TPC1 by NAADP.
• The S4–S5 linker emerges as a potential common determinant for TPC activation.

## Graphical abstract

**Figure:**
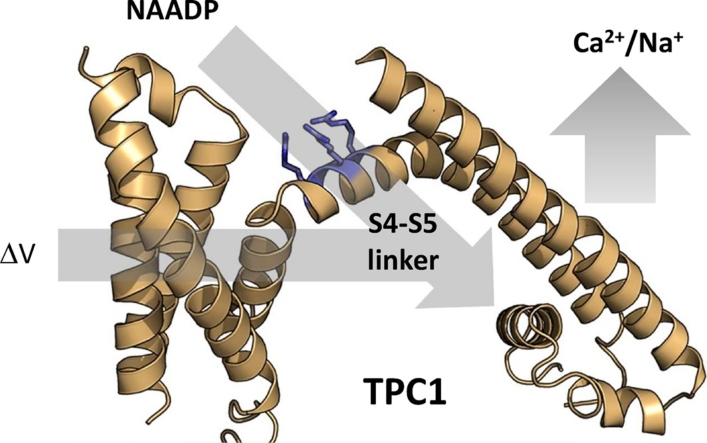

## 1 Introduction

TPCs are ancient members of the voltage-gated ion channel superfamily [1] with widespread physiological roles ranging from germination and stomatal movement in plants [2] to receptor and virus trafficking in animals [3], [4], [5]. They are structurally characterized by two homologous domains each comprising six trans-membrane regions (S1–S6) organized as a voltage sensing module (S1–S4) connected to the pore (S5–S6) by a cytosolic linker [6], [7]. TPCs assemble as dimers [8] and are likely evolutionary intermediates between tetrameric one domain and monomeric four domain channels exemplified by voltage gated K^+^ and Ca^2+^ channels, respectively [9].

Although it is established that TPCs localize to acidic organelles such as and plant vacuoles and animal lysosomes [10], [11], their mechanism of activation and ion selectivity are unclear [12], [13]. Plant TPC1 encodes the SV channel which is a vacuolar non-selective Ca^2+^-permeable channel regulated by voltage and Ca^2+^ [2]. Recent X-ray crystal structures have illuminated molecular mechanisms underlying voltage sensing and Ca^2+^ regulation [14], [15], [16]. But whether plant TPCs contribute to physiological cellular Ca^2+^ signals is debated [17]. Similarly, animal TPCs (encoded by up to 3 genes) were originally functionally identified as Ca^2+^-permeable endo-lysosomal ion channels activated by NAADP [18], [19], [20], [21], [22]. NAADP is a potent Ca^2+^ mobilizing messenger produced in response to numerous physiological stimuli such as hormones and neurotransmitters and long known to release Ca^2+^ from acidic organelles [23], [24], [25], [26]. But other studies suggest that TPCs are NAADP-insensitive Na^+^ channels regulated by the endo-lysosomal phosphoinositide, PI(3,5)P_2_ [27] and/or voltage [28], [29]. Evidence for [30] and against [31] the original findings continues to accrue. Little at present is known concerning the molecular determinants of TPC activation in animals.

Here, we identify arginine residues within the first S4-S5 linker of TPC1 critical for NAADP-evoked Ca^2+^ signalling and propose that this region is a common determinant for channel activation.

## 2 Methods

### 2.1 Bioinformatics

Human TPC1 (accession: AAI50204.1) was modelled based on *Arabidopsis thaliana* TPC1 (PDB:5E1J) using Phyre2 [32] in intensive mode and presented using the PyMOL Molecular Graphics System, Version 1.8 (Schrödinger, LLC). Multiple sequence alignments were performed using T-Coffee [33], Clustal Omega [34] and MUSCLE [35].

### 2.2 Plasmids

Constructs encoding human TPC1 tagged at its C-terminus with GFP or mRFP were described in [20]. Site-directed mutagenesis of TPC1-mRFP was performed using QuikChange (Stratech).

### 2.3 Other methods

Culture of SKBR3 cells, transfection, confocal microscopy, Ca^2+^ imaging and microinjection were performed as described in [36].

## 3 Results and discussion

### 3.1 The first S4-S5 linker of TPCs harbours conserved basic residues

**Fig. 1:**
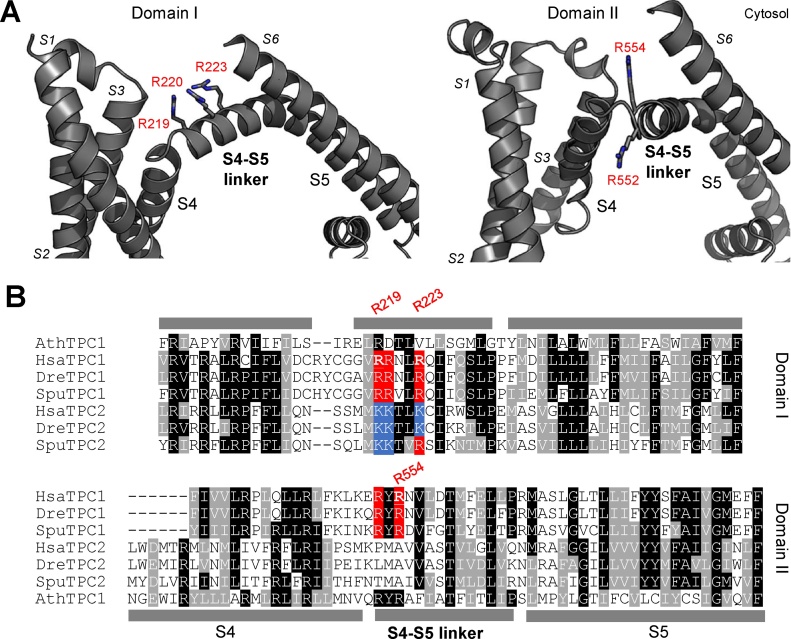
The first S4-S5 linker of TPCs harbours conserved basic residues. A. Structural model of human TPC1. Arginine residues in the S4-S5 linker of domain I (left) and domain II (right) are shown as sticks. B, T-Coffee multiple sequence alignment of TPC1 and TPC2 from *Arabidopsis thaliana* (Ath), *Homo sapiens* (Hsa), *Danio rerio* (Dre) and *Stronglyocentrotus purpuratus* (Spu). Conserved arginine (red) and lysine (blue) residues within the S4-S5 linkers of animal TPCs are highlighted. Arginine residues subjected to site-directed mutagenesis in human TPC1 are numbered. Secondary structure refers to that of AthTPC1. (For interpretation of the references to colour in this figure legend, the reader is referred to the web version of this article.)

Previous work concluded that human TPC1 but not TPC2 is a voltage-gated Na^+^ channel that confers endo-lysosomal excitability through arginine residues within putative voltage sensors [28]. S4 helices are established in mediating voltage sensing in a number of voltage-gated ion channels including plant TPC1 [14]. Taking advantage of recent plant TPC1 crystal structures [14], we modelled human TPC1 (Fig. 1A). Inspection of domain I revealed that R219, R220 and R223, which upon combined mutation eliminated voltage sensitivity [28] do not map to S4 (Fig. 1A). Instead, the residues were located downstream in the cytosolic linker connecting S4 to S5 (Fig. 1A–B).

Multiple sequence alignment showed that these residues were conserved mostly as lysine in human, zebrafish and sea urchin TPC2 (voltage-insensitive; Fig. 1B). Similar results were obtained using two additional alignment algorithms and a number of other species (data not shown). Our alignment agrees with those in [14], [15].

Together, this reappraisal suggests that the basic cluster is unlikely to directly mediate voltage-sensing by TPC1 and point to a more generic role in TPC function across isoforms.

### 3.2 Arginine residues in the first S4–S5 linker are required for activation of TPC1 by NAADP

**Fig. 2:**
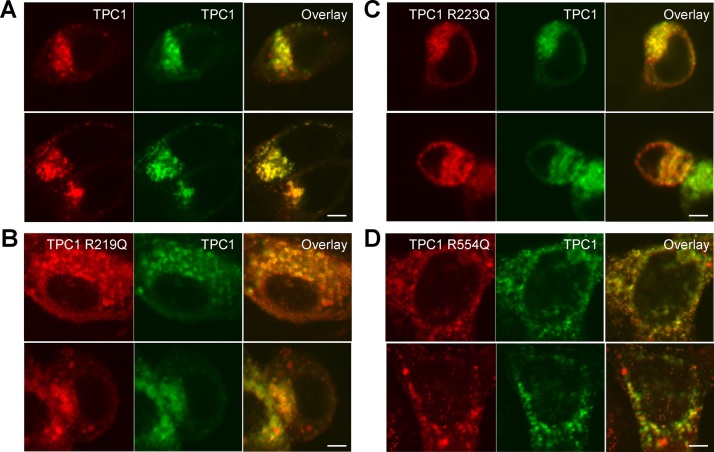
TPC1 mutated in the S4-S5 linkers express. Confocal images of SKBR3 cells expressing GFP-tagged TPC1 (middle panels) together with mRFP-tagged TPC1 (A), TPC1 R219Q (B), TPC1 R221Q (C) and TPC1 R554Q (D, left panels). Image overlays are shown to the right. Scale bars 2 μm.

**Fig. 3:**
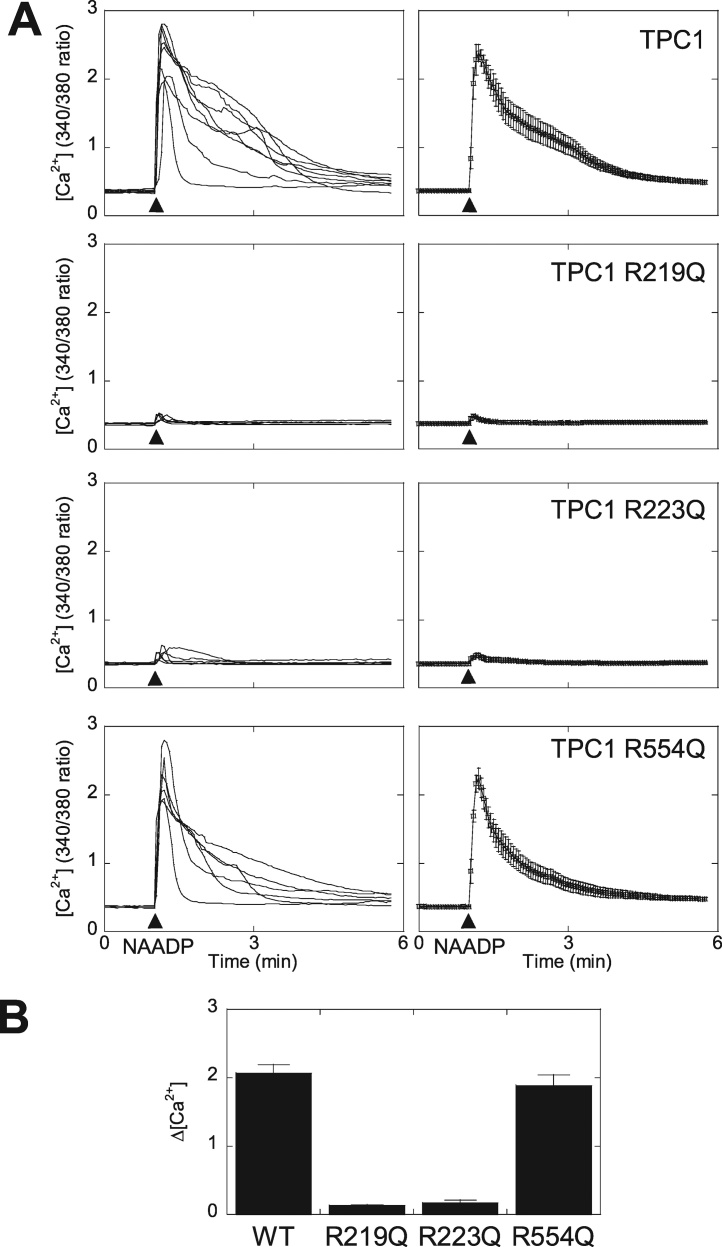
Arginine residues in the first S4-S5 linker are required for activation of TPC1 by NAADP. A, Cytosolic Ca^2+^ responses of cells expressing the indicated mRFP-tagged TPC1 construct and microinjected with NAADP (10 μM, pipette concentration). Responses of individual cells (left) and the average response of all cells are shown (right). B, Pooled data summarizing the change in [Ca^2+^]. Data are presented as mean ± S.E.M (n = 6–8).

To examine the functional role of the S4–S5 linker, we examined the consequences of mutating conserved arginine residues on Ca^2+^ release by NAADP in intact cells. TPC1 constructs in which either R219 or R223 had been mutated individually to glutamine expressed and colocalized with wild type TPC1 (Fig. 2A–C). Microinjection of NAADP evoked robust Ca^2+^ responses in cells expressing wild type TPC1 (Fig. 3A). The responses however were inhibited in cells expressing either mutant (Fig. 3A).

We also mutated R554 in the S4–S5 linker of domain II. This residue together with R552 is conserved in TPC1 but not TPC2 across species, and like the conserved residues in domain I, is placed within a helix (Fig. 1). Again, the mutant channel expressed and co-localised with wildtype TPC1 (Fig. 2D). However, in contrast to the domain I mutants, this construct supported NAADP-evoked signals (Fig. 3A).

Collectively, these data summarised in Fig. 3B, identify novel molecular determinants underlying NAADP action and further link TPCs to Ca^2+^ fluxes in intact cells.

### 3.3 Conclusions

In sum, prompted by new structural data, we identify a domain-specific requirement for the first S4-S5 linker in activation of TPCs by NAADP. Together with previous work implicating this same region in voltage-sensing [28] identifies it as a key mediator of TPC functionality. This region is unlikely to directly sense voltage (due to its placing out of the membrane) or bind NAADP (which interacts with accessory proteins [37]). Given the conservation of positive charge in TPC1 and TPC2, we speculate that mutations within the linker may instead affect channel activity indirectly through perturbing electrostatic interactions. This could be an intramolecular (allosteric) defect or an intermolecular one that disrupts association with putative NAADP binding proteins or anionic lipids such as PI(3,5)P_2_, which regulates both TPC isoforms [27], [28], [36]. Further analyses of this region across isoforms may rationalize multimodal activation of TPCs.

## Author contributions

EB performed the Ca^2+^ imaging. DC performed the confocal microscopy. SP designed the study, performed the bioinformatics and molecular biology, and wrote the paper.

## Competing financial interests statement

The authors declare that they have no competing interests.
